# Sugar Modification Enhances Cytotoxic Activity of PAMAM-Doxorubicin Conjugate in Glucose-Deprived MCF-7 Cells – Possible Role of GLUT1 Transporter

**DOI:** 10.1007/s11095-019-2673-9

**Published:** 2019-07-31

**Authors:** Krzysztof Sztandera, Paula Działak, Monika Marcinkowska, Maciej Stańczyk, Michał Gorzkiewicz, Anna Janaszewska, Barbara Klajnert-Maculewicz

**Affiliations:** 1 Department of General Biophysics, Faculty of Biology and Environmental Protection, University of Lodz, 141/143 Pomorska St, 90–236, Lodz, Poland; 2 Department of Surgical Oncology, Cancer Center, Copernicus Memorial Hospital, 62 Pabianicka St, 93–513, Lodz, Poland; 3 Leibniz–Institut für Polymerforschung Dresden e.V, 6 Hohe St, 01069 Dresden, Germany

**Keywords:** doxorubicin, glucose, glucose transporters, PAMAM dendrimer, tumor targeting

## Abstract

**Purpose:**

In order to overcome the obstacles and side effects of classical chemotherapy, numerous studies have been performed to develop the treatment based on targeted transport of active compounds directly to the site of action. Since tumor cells are featured with intensified glucose metabolism, we set out to develop innovative, glucose-modified PAMAM dendrimer for the delivery of doxorubicin to breast cancer cells.

**Methods:**

PAMAM-dox-glc conjugate was synthesized and characterized by ^1^H NMR, FT-IR, size and zeta potential measurements. The drug release rate from conjugate was evaluated by dialysis under different pH conditions. The expression level of GLUT family receptors in cells cultured in full and glucose-deprived medium was evaluated by quantitative real-time RT-PCR and flow cytometry. The cytotoxicity of conjugate in presence or absence of GLUT1 inhibitors was determined by MTT assay.

**Results:**

We showed that PAMAM-dox-glc conjugate exhibits pH-dependent drug release and increased cytotoxic activity compared to free drug in cells cultured in medium without glucose. Further, we proved that these cells overexpress transporters of GLUT family. The toxic effect of conjugate was eliminated by the application of specific GLUT1 inhibitors.

**Conclusion:**

Our findings revealed that the glucose moiety plays a crucial role in the recognition of cells with high expression of GLUT receptors. By selectively blocking GLUT1 transporter we showed its importance for the cytotoxic activity of PAMAM-dox-glc conjugate. These results suggest that PAMAM-glucose formulations may constitute an efficient platform for the specific delivery of anticancer drugs to tumor cells overexpressing transporters of GLUT family.

**Electronic supplementary material:**

The online version of this article (10.1007/s11095-019-2673-9) contains supplementary material, which is available to authorized users.

## Introduction

The incidence of cancer is one of the biggest problems of public health. It is expected that due to the constant epidemiological and demographic changes, the number of cancer cases will continuously increase over the next decades (1). Breast cancer is one of the most commonly occurring tumors among women in highly developed countries. According to World Health Organization (WHO), there are about 1.5 million new cases of breast cancer each year. Different variants of breast cancer are characterized by high heterogeneity and diverse metastasis mechanisms, which constitute significant obstacles for the development of an effective therapy (2,3).

Several methods of breast cancer treatment may be distinguished, including chemotherapy, radiotherapy, hormone therapy, targeted therapy and surgery. Typically, the treatment involves a combination of these methods (4). The choice of therapy depends on the stage and the advancement of the disease, as well as patient’s overall condition. Among the strategies of cancer treatment, chemotherapy is most commonly used, but in many cases, it is not sufficient. Its effectiveness may be limited by unfavorable biodistribution of anticancer drugs or their low solubility, but most importantly – by low specificity of action of these therapeutics, which leads to various side effects and systemic toxicity. Changeable microenvironment and drug resistance of cancer cells additionally hamper the elaboration of efficient therapy (5).

As the development of novel anticancer compound and its commercialization is extremely expensive, time-consuming and labor-intensive, the interest of scientists turned towards drug carrier systems, aiming at improvement of the efficacy of established chemotherapy, reduction of detrimental side effects and overcoming the multidrug resistance. Modern methods of nanotechnology allow manufacturing of compounds with wide range of applications in drug delivery, from single nanoparticles to more complex polymers and macromolecules (6). Nanostructures have been found to protect the therapeutics from degradation, enhance their solubility, prolong blood half–life and provide targeted delivery and controlled release of active compounds (7), allowing to circumvent several limitations of classical anticancer treatments. To date, a number of nanoparticles, including liposomes, nanotubes and dendrimers have been evaluated for their drug delivery potential (8).

Numerous studies indicate that highly-branched, monodisperse dendrimers of well-defined structure are the most valid choice for drug carriers. The nanometric size and globular shape of dendrimers favor their cellular uptake, at the same time decreasing their renal filtration rate and extending circulation time (9). High molecular weight additionally promotes tumor localization of dendrimer macromolecules due to the so-called enhanced permeability and retention (EPR) effect (10).

The dendrimers’ three-dimensional architecture with reactive terminal groups and numerous internal cavities gives the possibility to physically entrap the therapeutics inside the dendritic scaffold or bind them on the surface of macromolecule (either covalently or non-covalently, due to the electrostatic or van der Waals interactions) (11). The application of drug-dendrimer covalent conjugates seems to be the most convenient approach. Appropriate selection of the linker and creation of permanent bonding may provide superior stability and improved controlled release patterns compared to non-covalent complexes (12). Furthermore, dendrimers may be surface-modified with molecules specifically interacting with cancer cells, additionally improving the targeting potential of such drug delivery devices (13).

Tumor microenvironment is characterized by variable and heterogeneous concentration of compounds and nutrients, especially glucose. Glucose deficiency rapidly occurs in the inner mass of growing tumor, resulting from atypical blood vessel formation, defective blood perfusion and unrestricted cancer cell proliferation. Thus, cancer cells undergo specific changes in their metabolism, including increased uptake of glucose (14). Further, the occurrence of Warburg effect is a characteristic feature of cancer cells. This phenomenon is associated with high glycolysis rates, even under aerobic conditions. As a consequence, cancer cells consume more glucose during growth and produce higher amounts of lactate compared to normal cells (15). The exploitation of these anomalies in cancer therapy arouses the interest of researchers, particularly in case of specific delivery of therapeutics. One of the promising approaches involves glycoconjugation, i.e. binding of an anticancer drug with glucose or different sugar, which may increase the cellular uptake of the therapeutic compound (16).

Taking all above-mentioned aspects into consideration, we set out to evaluate the potential of poly(amidoamine) (PAMAM) dendrimers for the improved delivery of doxorubicin, one of the most popular anticancer drugs. For the enhancement of PAMAM-doxorubicin cytotoxic activity, the dendrimer surface was modified with glucose molecule, in order to utilize glucose uptake-dependent pathway for the specific and more efficient delivery of the drug into breast cancer cells.

## Materials and Methods

### Materials

All chemical reagents were purchased from commercial suppliers. Solvents for the synthesis were purchased from Sigma-Aldrich. All cell culture reagents were purchased from Gibco® (Germany). Flasks and multiwell plates for *in vitro* studies were obtained from Nunc (Germany). Amine terminated PAMAM G4 dendrimer, doxorubicin hydrochloride, PBS (phosphate buffered saline), FBS (fetal bovine serum) and MTT (3-[4,5-dimethylthiazol-2-yl]-2,5-diphenyltetrazolium bromide) were purchased from Sigma-Aldrich. Trypan blue was purchased from Molecular Probes (USA). Caelyx® (PEGylated liposomal doxorubicin) was purchased from Janssen-Cilag International (USA). Rubusoside and kaempferol were purchased from Sigma-Aldrich. Human breast adenocarcinoma cell line MCF-7 (ATCC no. HTB-22) was purchased from ATCC (USA). AntiGLUT1-FITC antibody (FAB1418F) was purchased from R&D.

### Synthesis of PAMAM-Doxorubicin and PAMAM-Doxorubicin-Glucose Conjugates

The synthesis of PAMAM-doxorubicin (PAMAM-dox, molar ratio 1:1) conjugate was performed as described previously (17). Briefly, doxorubicin (5.8 mg, 10 μmol) was dissolved in 5 ml of 0.1 M PBS at 25°C. *Cis*–aconitic anhydride (CAA; 2.4 mg, 15 μmol) was dissolved in 500 μl of *p*–dioxane and slowly added to the doxorubicin solution while maintaining the reaction mixture pH at 8.5. The solution was incubated with stirring for 20 min, then for 20 min at 25°C, in dark. Then the reaction mixture was cooled on ice and supplemented with 100 mM HCl until pH reached 3.0 and extracted by ethyl acetate. Glucose (1.8 mg, 10 μmol) was dissolved in 5 ml of 0.1 M PBS at 25°C. Succinic anhydride (SA; 1.5 mg, 15 μmol) and N-(3-Dimethylaminopropyl)-N′-ethylcarbodiimide hydrochloride (EDC; 3.8 mg, 20 μmol) were slowly added to glucose solution. The reaction mixture was stirred at room temperature for 2 h. In the second step dox-CAA was used to produce PAMAM-dox conjugate or dox-CAA and glucose-SA to produce PAMAM-doxorubicin-glucose (PAMAM-dox-glc, molar ratio 1:1:1) conjugate. To the resulting dox-CAA and glucose-SA in PBS (pH 6), 5-fold molar excess of EDC was added, and the mixture was stirred at 20°C for 0.5 h in dark. PAMAM G4 was dissolved in 1 ml of PBS, pH 6.0 and dox-CAA was added. The mixture was incubated with intensive stirring at 25°C, pH 7.8 for 12 h. PAMAM-dox-glc was purified by ultrafiltration on an Amicon Ultra-3 K (molecular weight cut-off, MWCO 3 kDa). ^1^H NMR and FT-IR were used to confirm the purity of the products and to ascertain the level of conjugation. ^1^H NMR spectra were recorded on Bruker Avance III DRX-600 and 500 MHz spectrometers, using D_2_O as solvent. The FT-IR spectra were collected with a FT-IR ATI Mattson Spectrometer Spectrum and samples were measured as thin film in KBr crystals. The number of scans was 32. All spectra were collected in the range (4000–400 cm^−1^).

### Size and Zeta Potential of PAMAM and PAMAM-Doxorubicin-Glucose Conjugate

Measurements of size and zeta potential were performed with the use of Zetasizer Nano ZS (Malvern Instruments Ltd., UK). Water solutions containing studied compounds in a final dendrimer concentration of 10 μM were placed in the low volume sizing cuvettes (ZEN0112, Malvern) for size determination or in the folded capillary cells (DTS 1070, Malvern) for zeta potential measurements and measured at 25°C. The data were analyzed using the Malvern software.

### pH-Dependent Release of Doxorubicin from PAMAM-Doxorubicin-Glucose Conjugate

In order to evaluate the rate of doxorubicin release from the conjugate in different pH conditions, 0.5 μM solution of PAMAM-doxorubicin-glucose conjugate in PBS (pH 7.5) was enclosed in a dialysis membrane tubing (SnakeSkin™ Dialysis Tubing, 3.5 K MWCO, 22 mm, ThermoFisher) and immersed in PBS pH 5.5, 6.5 or 7.5. Samples from the external phase were collected at subsequent intervals (1, 3, 6, 24 and 48 h) and the doxorubicin fluorescence at 480 nm excitation and 590 nm emission was measured on PowerWave HT Microplate Spectrophotometer (BioTek, USA).

### Cell Culture

Human breast adenocarcinoma (MCF-7) cell line was grown in DMEM medium with different glucose concentration (0 or 4.5 g/L) and 10% (*v/v*) fetal bovine serum (FBS). Cells were cultured in T-75 culture flasks at 37°C, 5% CO_2_ and subcultured every 2 or 3 days. Cells were harvested and used in the experiments after obtaining 80–90% confluence. The number of viable cells was determined by trypan blue exclusion assay with the use of Countess Automated Cell Counter (Invitrogen).

### Cytotoxicity Assay

The influence of PAMAM dendrimer conjugates and liposomal doxorubicin Caelyx® on the cell viability was determined with the use of the MTT assay. Briefly, different concentrations of all compounds were added to the 96–well plates containing cells at the density of 2.0 × 10^4^ cells per well in medium. Cells were incubated with compounds for 24 and 48 h at 37°C, 5% CO_2_. After the incubation period cells were washed once with PBS, 50 μL of a 0.5 mg/mL solution of MTT in PBS was added to each well and cells were further incubated under normal culture conditions for 3 h. After incubation the MTT solution was removed and the obtained formazan precipitate was dissolved in DMSO (100 μL per well). The conversion of the tetrazolium salt (MTT) to a colored formazan by mitochondrial and cytosolic dehydrogenases is a marker of cell viability. Before the measurement plates were shaken for 1 min and the absorbance at 570 nm was measured on PowerWave HT Microplate Spectrophotometer (BioTek, USA).

For the inhibition of GLUT1-related cellular uptake, cells were pretreated with rubusoside (5 mM) or kaempferol (100 μM) for 1 h, then the conjugates were added at the final concentration of 10 μM. Cells were subsequently incubated with tested compounds for 24 and 48 h at 37°C, 5% CO_2_. After the incubations MTT assay was performed.

### Gene Expression Assay

The gene expression level was determined by quantitative real-time RT-PCR. MCF-7 cells cultured in medium containing 0 or 4.5 g/L of glucose were seeded into 6-well places (7 × 10^5^ cells per well) and incubated for 24 h at 37°C, 5% CO_2_. To examine the effect of the inhibitor on GLUT1 expression, an additional 6 h incubation of MCF-7 cells with 100 μM kaempferol was performed. Following incubation, total cellular RNA was isolated using TRI Reagent (Sigma-Aldrich) according to manufacturer’s protocol. Complementary DNA (cDNA) was transcribed from mRNA using M–MLV Reverse Transcriptase system (Promega) and used for real-time PCR amplification with the GoTaq® qPCR Master Mix (Promega) according to manufacturer’s protocol. Each 16 μl reaction volume contained 1 μl of cDNA and 0.25 μM of forward and reverse intron-spanning primers (for primer sequences, see Table I). The reference genes (*HPRT1* and *TBP*) were selected according to the GeNorm procedure (18). PCR reactions were performed in 96-well microplates using the CFX96 Real Time PCR Detection System (Bio-Rad). The expression level of assayed genes was calculated by the ΔCt method and expressed as number of cognate mRNA copies per 1000 copies of geometric–averaged mRNA for reference genes.

**Table I:** Primer Sequences

Transporter	Gene name	Forward and reverse sequences (5′–3′)
	*HPRT1*	Fw: TGACACTGGCAAAACAATGCA
		Rv: GGTCCTTTTCACCAGCAAGCT
	*TBP*	Fw: CACGAACCACGGCACTGATT
		Rv: TTTTCTTGCTGCCAGTCTGGAC
GLUT1	*SLC2A1*	Fw: GGGCCAAGAGTGTGCTAAAG
		Rv: GTTGACGATACCGGAGCCAA
GLUT3	*SLC2A3*	Fw: GACCCAGAGATGCTGTAATGGT
		Rv: TTATGATCTTCTCAGGAGCATTGA
GLUT4	*SLC2A4*	Fw: CCTCTCCGTGGCCATCTTTT
		Rv: GATGTCAGCCCTGAGTAGGC
GLUT5	*SLC2A5*	Fw: GGGGCACCCACTTACTTAGC
		Rv: CACGTTGGAAGATACACCCCTT
GLUT12	*SLC2A12*	Fw: ACTGTAACTGATCTTATTGGCCTG
		Rv: GATTGGCCCCTACCACACAG

### Flow Cytometry Analysis

GLUT1 surface expression was assessed using flow cytometry. MCF-7 cells were seeded into 12-well plates (2.5 × 10^5^ cells per well). After 24 h of incubation (37°C, 5% CO_2_), the medium was removed from the wells and the inhibitors (rubusoside (5 mM) or kaempferol (100 μM)) dissolved in fresh medium were added. The plates were incubated for 6 h (37°C, 5% CO_2_), the medium was removed from the wells and 150 μL of trypsin was applied to separate the cells from the bottom of the plate. After 5 min, the trypsin was neutralized by adding 350 μL of the appropriate medium. The cells were then washed with 4 mL of medium. After centrifugation at 1000×g for 5 min the supernatant was decanted and the pellets were resuspended in 2 mL of PBS, then centrifuged three times 500×g for 5 min and rinsed each time with PBS to remove all remaining impurities. After washing, the cells were transferred to cytometric tubes (2.5 × 10^5^ cells in 100 μL per tube). Then, 20 μL of antiGLUT1-FITC antibody was added to each tube, and the cells were incubated for 30 min at room temperature in dark. The cells were subsequently washed three times with PBS, resuspended in 400 μL of PBS and analyzed on LSR II system (Becton Dickinson, USA). In each experiment 10000 events were counted after gating of viable cells and median of fluorescence intensity in FITC channel was calculated for each population. As a control, unlabeled cells were measured similarly and median of auto-fluorescence intensity was calculated and subtracted from each experimental result.

### Statistical Analysis

Analysis of variance (ANOVA) with the Tukey’s post hoc test was used for results comparison. For single pairwise comparisons, Student’s t test was applied. All statistics were calculated using the Statistica software (StatSoft, Tulsa, USA), and *p* values <0.05 were considered significant.

## Results and Discussion

### Synthesis and Characterization of PAMAM-Doxorubicin and PAMAM-Doxorubicin-Glucose Conjugates

**Fig. 1:**
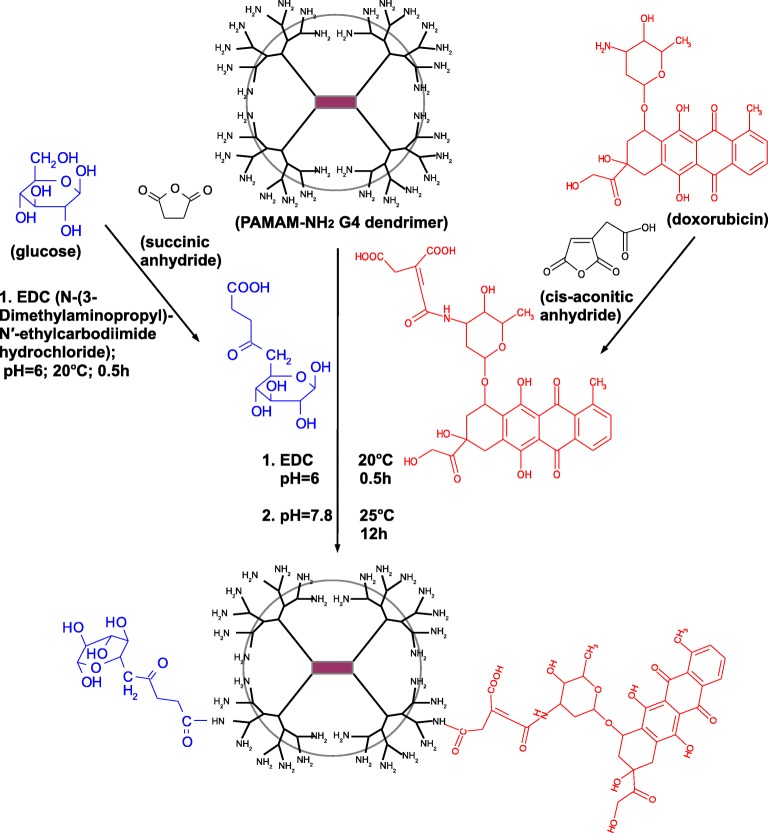
Scheme of PAMAM-doxorubicin-glucose (PAMAM-dox-glc) conjugate synthesis.

**Fig. 2:**
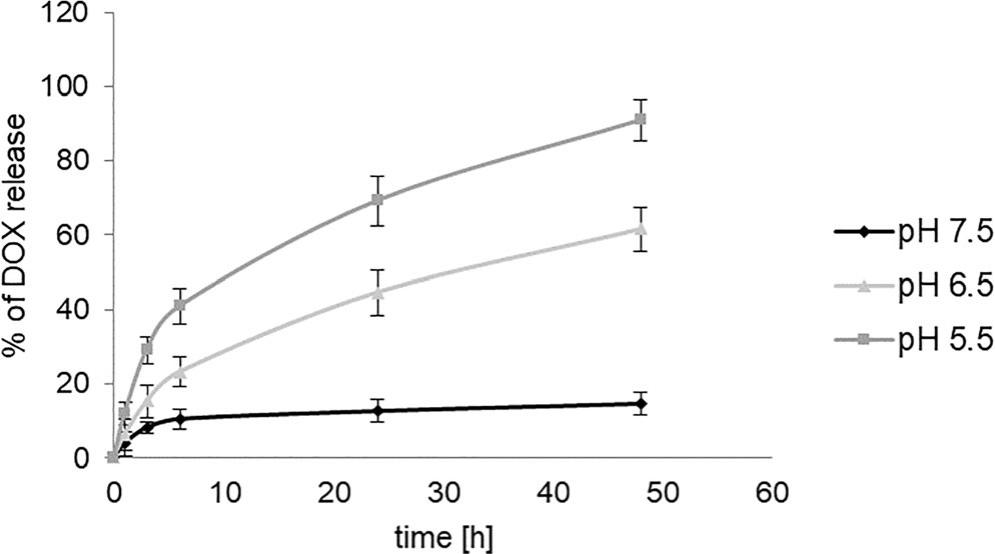
The release of doxorubicin from PAMAM-dox-glc conjugate in different pH conditions, determined by dialysis. Data presented as average ± SD.

The main disadvantage of most cancer treatments involves low specificity of the drugs towards cancer cells, leading to high systemic toxicity and numerous side effects. In order to overcome this obstacle, we synthesized the conjugate consisting of three components (Fig. 1 and Figs. S1-S7 in Supplementary Materials), each of them meant to play a different role: (1) glucose as a vector providing effective transport of the conjugate to the cancer cells, (2) doxorubicin responsible for the cytotoxic effect, (3) PAMAM dendrimer bound with doxorubicin via pH-sensitive linker (cis-aconitic anhydride) undergoing hydrolysis in acidic pH (Fig. 2 and (19)), thus protecting the whole conjugate in the circulatory system and enabling controlled delivery and specific drug release in the tumor environment.

Doxorubicin and glucose conjugation increased the diameter and lowered zeta potential of the conjugate in comparison to unmodified PAMAM dendrimer (Table II), however the conjugate retained the nanometric size and overall surface positive charge, favoring efficient endocytosis and cell entry (20).

**Table II:** Size and Zeta Potential Of PAMAM Dendrimer and PAMAM-Dox-Glc Conjugate. Data Presented as Average ± SD

	PAMAM	PAMAM-dox-glc
diameter [nm]	3.83 ± 0.34	12.93 ± 3.51
zeta potential [mV]	23.81 ± 3.24	14.26 ± 1.54

### Cytotoxicity of Nanocarriers

**Fig. 3:**
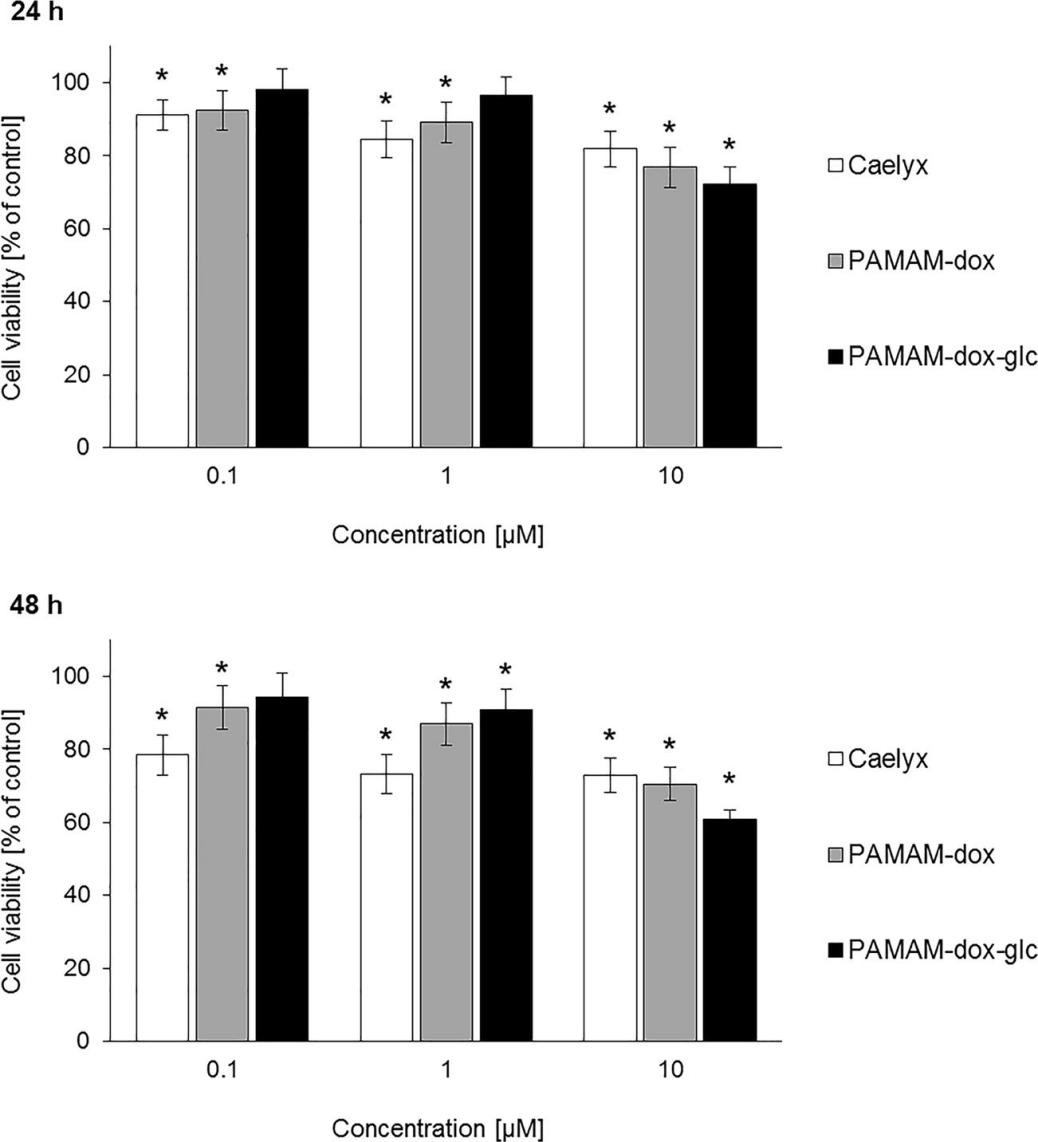
The effect of tested compounds on the viability of MCF-7 cells cultured in complete medium. * *p* < 0.05 relative to the control. Data presented as average ± SD.

Cytotoxicity of Caelyx®, PAMAM-doxorubicin (PAMAM-dox) and PAMAM-doxorubicin-glucose (PAMAM-dox-glc) conjugates were assessed with MTT assay. In the concentration range of 0.1–10 μM, comparable toxicity of tested compounds towards MCF-7 cells grown in a complete medium (4.5 g/L glucose) was observed after 24 and 48 h of incubation (Fig. 3). Taking into account the specific metabolism of cancer cells, in the further course of our research MCF-7 cell line cultured in medium without glucose was applied.

**Fig. 4:**
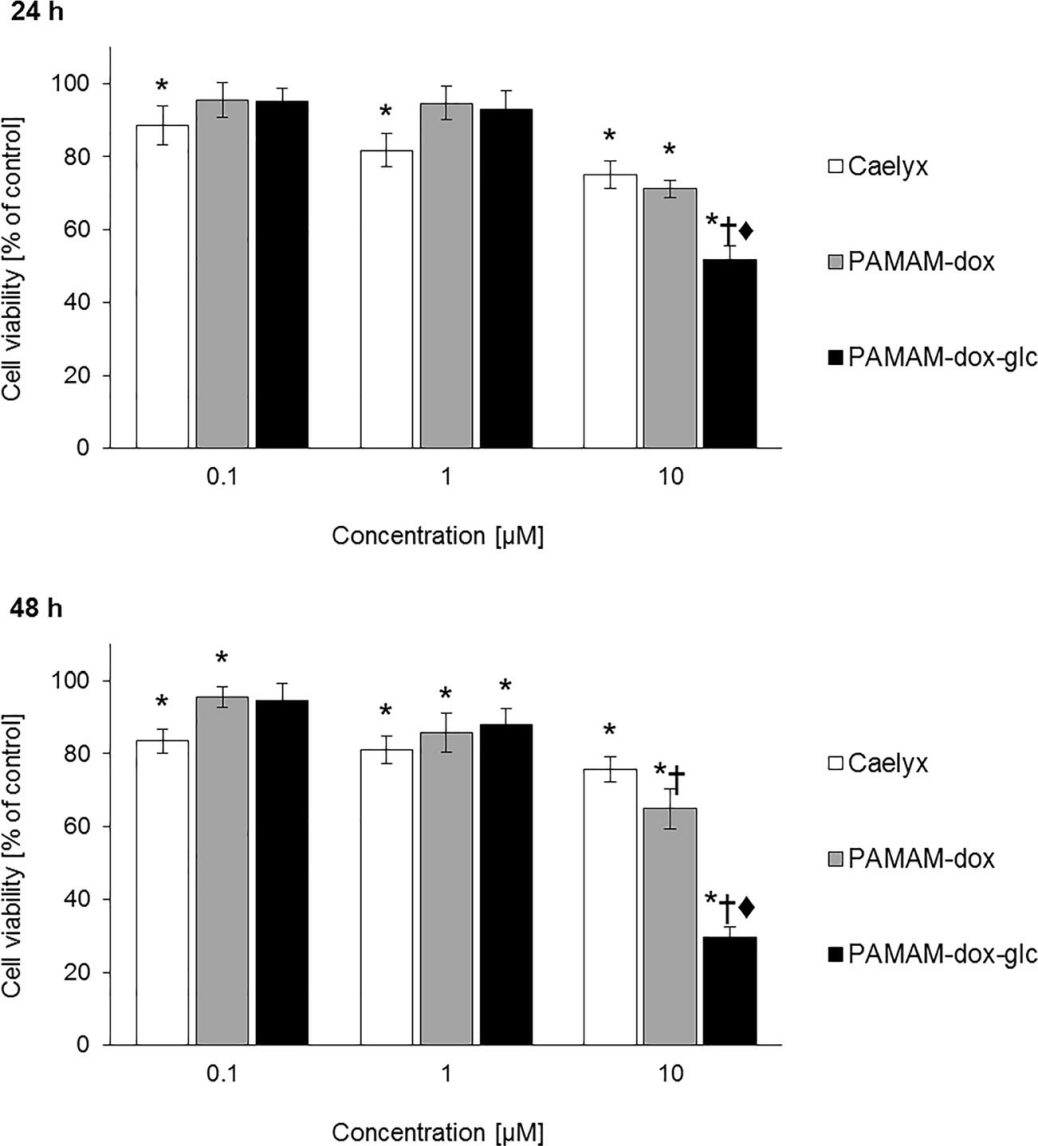
The effect of tested compounds on the viability of MCF-7 cells cultured in medium without glucose. * *p* < 0.05 relative to the control; † *p* < 0.05 relative to Caelyx®; ♦ *p* < 0.05 relative to PAMAM-dox. Data presented as average ± SD.

As expected, complete removal of glucose from the culture medium increased the cytotoxicity of PAMAM-dox-glc conjugate at the highest concentration tested compared to Caelyx® and PAMAM-dox, both after 24 and 48 h of treatment (Fig. 4).

Strongly increased toxicity of PAMAM-dox-glc conjugate in glucose-deprived MCF-7 cells may be attributed to the occurrence of the Warburg effect, involving high rate of glucose uptake and aerobic glycolysis followed by lactic acid fermentation in cancer cells (15). Therefore, we postulate that glucose-conjugated dendrimer is taken up by the cells more rapidly in the absence of sugar source.

Our hypothesis and observations for PAMAM-dox-glc conjugate being more effective than free drug find support in several previous studies indicating that glycoconjugation may provide specific delivery of therapeutics to cancer cells. Numerous reviews on carbohydrate-drug complexes have been published concerning the role of glycoconjugated therapeutics in the treatment of cancer. Saccharide-conjugated analogs of diverse agents, designed to improve water solubility, serum stability and targeting properties of therapeutics, have been reported in the scientific literature since the early 1990s. Currently several compounds possessing sugar moiety are under evaluation both *in vitro* and *in vivo* for the application in anticancer therapy. These include DNA break-inducing and intercalating agents, alkylating compounds, nitrogen mustards and heavy metals. Such formulations have been shown to provide enhanced cytotoxicity and specific tumor accumulation, at the same time reducing systemic toxicity (16,21,22). These observations, together with the results of our experiments indicate that sugar moieties may constitute efficient targeting molecules for specific delivery of drugs and nanocarriers to cancer cells.

### Expression of SLC2 (GLUT) Membrane Transporters

Taking the MMT assay results into consideration, we hypothesized that the enhanced toxicity of PAMAM-dox-glc in glucose-deprived MCF-7 cells may be associated with increased expression of glucose transporters of SLC2 (GLUT) family. Although the regulation of GLUTs by substrate availability is poorly characterized, several reports indicate the down-regulation of GLUT gene expression by high concentration of glucose (23). Thus, we applied real-time RT-PCR to assess the expression of GLUT coding SLC2 genes (Table III).

**Table III:** The Relative Expression Level of GLUT Family Genes in MCF-7 Cells Cultured in Media with Different Glucose Concentration (0 or 4.5 g/L). The Expression was Evaluated Using Quantitative Real-Time RT-PCR. Data Expressed as Relative mRNA Copy Number Per 1000 Copies of Averaged Reference mRNA, Calculated by 2^–∆Ct^ Transformation, Presented as Average ± S.E.M, *n* = 3. * Indicate Statistically Significant Difference Between Samples, *p* < 0.05

	GLUT1	GLUT3	GLUT4	GLUT5	GLUT12
0 g/L	3508.09 ± 165.31	22.95 ± 11.31	109.33 ± 11.28	223.08 ± 14.93	1483.03 ± 52.18
4.5 g/L	984.07 ± 409.12	13.69 ± 2.78	24.29 ± 6.46	0.03 ± 0.02	55.14 ± 19.91
	*		*	*	*

As expected, MCF-7 cells cultured in glucose-free medium exhibited higher expression of GLUT transporters, exceeding the expression in cells grown in complete medium by 3.6-fold for GLUT1, 4.5-fold for GLUT4, 11 × 10^3^-fold for GLUT5 and 27-fold for GLUT12. For the expression of GLUT3 gene, statistically significant difference was not observed.

It should be noted that the transport across cellular membrane mediated by surface glucose transporter (GLUT) proteins is the first rate-limiting step of glucose metabolism (24). The level of surface expression of GLUTs greatly influences the glucose uptake into the cells, and may have a significant impact on cellular glucose homeostasis (25). Each of the glucose transporter proteins possesses varied affinity for glucose and different carbohydrates. Extensively studied GLUT1, 3 and 4 are featured with highest affinity for glucose (26), with GLUT1 present at variable levels in many tissues, being responsible for basal glucose uptake (27). The specificity of recently discovered GLUT12 towards glucose is not fully known. GLUT12-mediated glucose transport can be competed with different substrates, like fructose, galactose or cytochalasin B (28). By contrast, GLUT5 has high affinity for fructose and low for glucose (29).

Overexpression of membrane glucose transporters belonging to GLUT family is a common feature of different malignancies (30), which may be exploited for targeted delivery of drug-nanoparticle conjugates via glucose uptake-dependent pathway (16). This strategy has been successfully applied previously for specific delivery of doxorubicin by glucose-conjugated chitosan nanoparticles, which showed 4–5 times increased endocytosis into GLUT overexpressing 4T1 cells compared to formulations without glucose (31). Further, glucose-coated superparamagnetic iron oxide nanoparticles showed higher internalization rate in BxPC3 pancreatic adenocarcinoma cell line expressing GLUT1 transporter than in normal MRC5 cells (32). Poly(ethylene glycol)-co-poly(trimethylene carbonate) nanoparticles modified with 2-deoxy-D-glucose (DGlu-NPs) were used for glioma therapy, targeting both the blood-brain barrier and the tumor, and exhibiting enhanced cellular uptake in RG-2 rat glioma cell line compared to the non-modified nanoparticles. Interestingly, the uptake of such nanoparticles was inhibited by free glucose, suggesting the involvement of GLUT1. Further, DGlu-NPs showed specific tumor accumulation and improved antiglioma efficiency *in vivo* (33), and D-glucosamine-modified nanoparticles targeting GLUT1 displayed significant antiglioma activity both *in vitro* and *in vivo* (34).

Since GLUT expression is frequently altered in human breast tumors (35), the idea of drug delivery associated with intracellular transport of carbohydrates may be especially promising for this type of cancer. For instance, Shan *et al.* synthesized γ-Fe_2_O_3_ nanoparticles coated with dimercaptosuccinic acid and functionalized with 2-deoxy-D-glucose (γ-Fe_2_O_3_@DMSA-DG NPs) to target them to GLUT1-overexpressing breast cancer, and observed significantly increased MRI signal intensity of MDA-MB-231 cells treated with those formulations (36). Fructose-coated micelles with varying sizes were found to be efficiently taken up by breast cancer cell lines (MCF-7 and MDA-MB-231) expressing GLUT5, more rapidly than in case of normal cells (CHO and RAW264.7) (37). The analysis of Venturelli *et al.* revealed that glucose shell of magnetic nanoparticles (MNPs) plays a key role in recognition by cells with rapid metabolism. Inhibition studies involving selective blocking of GLUT1 suggested that this protein is responsible for cellular uptake of glucose-coated MNPs. Interestingly, the concentration of glucose in culture medium was found to drive the internalization of these nanoparticles into MCF-7, decreasing it by 79.3% from normal to high glucose concentration (38). Recently, glycoconjugation strategy has been used for the mitochondrial delivery of paclitaxel with glucose-modified PAMAM dendrimers, which showed higher cellular uptake both in GLUT1-overexpressing MCF-7/MDR monolayer and multicellular tumor spheroids (39). All these observations indicate the great potential of GLUT-mediated cellular uptake of sugar-conjugated drug carriers and justify our idea for the intracellular delivery of anticancer drugs by glucose-modified PAMAM.

Our real-time RT-PCR assay showed that GLUT1 transporter gene is characterized by the highest expression among proteins of this family in MCF-7 cell line. Therefore, we decided to determine whether the two well-known inhibitors of GLUT1, rubusoside and kaempferol (40–42) may influence the expression of this transporter, and hence – the cytotoxic activity of PAMAM-dox-glc conjugate towards breast cancer cells.

Since kaempferol has been reported as mixed-type inhibitor, rapidly decreasing the activity of GLUT1 protein and additionally its mRNA expression during extended periods of incubation (41), we examined its impact both on the level of mRNA and active surface protein. After 6 h of incubation with kaempferol, 9- and 68-fold decrease in GLUT1 gene expression was observed in cells cultured in complete medium and medium without glucose, respectively (Table IV).

**Table IV:** The Relative Expression Level of GLUT1 Gene in MCF-7 Cells Cultured in Media with Different Glucose Concentration (0 or 4.5 g/L) after the Treatment with Kaempferol (100 μM). The Expression was Evaluated Using Quantitative Real-Time RT-PCR. Data Expressed as Relative mRNA Copy Number Per 1000 Copies of Averaged Reference mRNA, Calculated by 2^–∆Ct^ Transformation, Presented as Average ± S.E.M, *n* = 3. * Indicate Statistically Significant Difference Between Samples, *p* < 0.05

	0 g/L	4.5 g/L
– kaempferol	3508.09 ± 165.31	984.07 ± 409.12
+ kaempferol	51.10 ± 4.04	111.83 ± 4.12
	*	*

**Fig. 5:**
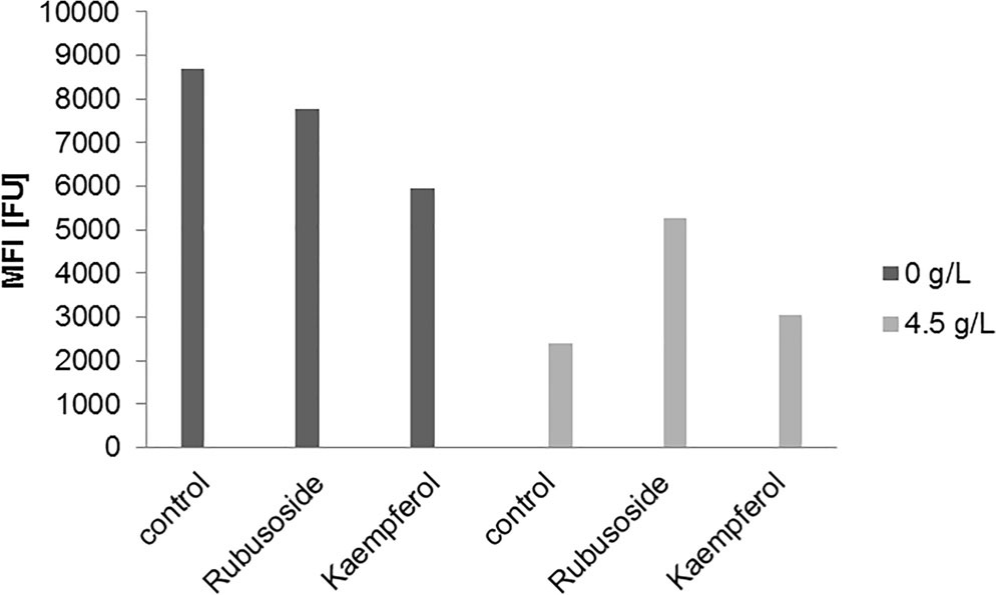
A representative results of flow cytometry experiments: GLUT1 surface protein expression in MCF-7 cells cultured in media with different glucose concentration (0 or 4.5 g/L).

In line with the outcome of real-time RT-PCR assay, cytometric analysis confirmed approximately 4-fold increase in expression of GLUT1 protein in MCF-7 cells cultured in glucose-deprived environment, compared to those grown in complete medium (Fig. 5). In addition, our results corroborated previous reports on the dual nature of kaempferol, which inhibited GLUT1 expression also on the protein level in cells in medium without glucose, showing stronger effect than rubusoside. In case of MCF-7 cultured in complete medium, the inhibitory effect on surface expression of GLUT1 protein was not observed for either rubusoside or kaempferol.

### Cytotoxicity of Conjugates in the Presence of GLUT1 Inhibitors

In order to determine the involvement of GLUT1 in the activity of PAMAM-dox-glc formulations, MCF-7 cells in glucose-deprived medium were treated with rubusoside or kaempferol. Subsequent evaluation of cell viability upon incubation with PAMAM-dox and PAMAM-dox-glc clearly indicated the vital role of GLUT1 in cellular uptake of glucose-modified nanocarrier.

**Fig. 6:**
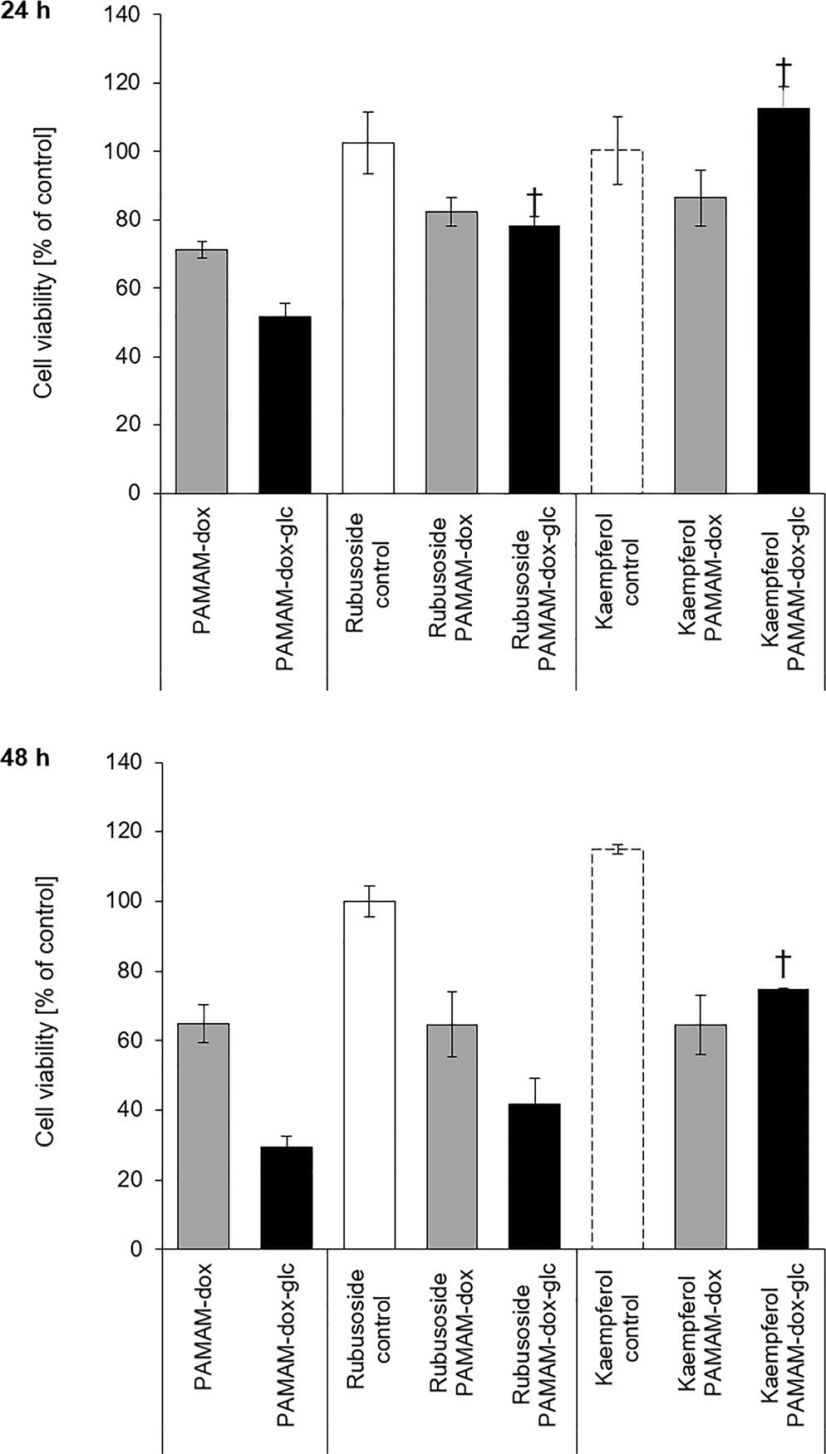
The effect of tested compounds on the viability of MCF-7 cells cultured in medium without glucose after treatment with GLUT1 inhibitors. † *p* < 0.05 relative to PAMAM-dox-glc without inhibitors. Data presented as average ± SD.

Neither rubusoside nor kaempferol affected the viability of glucose-deprived cells, at the same time notably limiting the cytotoxic activity of PAMAM-dox-glc conjugate. After 24 h of treatment, statistically significant differences were confirmed for both tested inhibitors (with rubusoside increasing cell viability from 50 to 80% after treatment with glucose-modified conjugate, and kaempferol completely eliminating the toxic effect of PAMAM-dox-glc). After 48 h of incubation, significant difference was observed only for cells pretreated with kaempferol (Fig. 6). Both inhibitors had almost no effect on the cytotoxicity of PAMAM-dox, proving our hypothesis on the crucial targeting role of glucose moiety and GLUT1-associated transport of PAMAM-dox-glc.

These results ultimately indicate the important role of GLUT1 in the transport of PAMAM-dox-glc conjugate: protein expression after the treatment with inhibitors in MCF-7 cells cultured without glucose is significantly reduced, which correlates with their increased survival after incubation with PAMAM-dox-glc in the presence of kaempferol/rubusoside. This may be further supported by the outcome of quantitative real-time PCR and Western blot experiments performed by Krzeslak *et al.*, which also points to the correlation between increased expression of GLUT1 gene and functional protein (43). Interestingly, the observations of Cantuaria *et al.* indicate a better response to chemotherapy in patients with tumors overexpressing for GLUT1, which may be associated not with the glucose transport itself but with accelerated metabolism of cancer cells, and as a consequence – higher activity of drugs that act specifically on rapidly dividing cells (44). This phenomenon additionally favors the idea of utilizing the overexpression of this receptor in targeted anticancer therapy.

Nevertheless, the proposed method of using GLUT1 overexpression for the specific delivery of PAMAM-dox-glc conjugate and the results of our experiments indicate that increased expression of glucose transporters may be a predisposing factor for enhanced response of cancer cells to chemotherapy with the use of glucose-modified drug carriers.

## Conclusions

The elaboration of tumor-specific drug delivery system based on receptor-mediated endocytosis is extremely challenging due to the biological complexity of tumors and their environment. One of the key features of cancer cells involves the up-regulation of glucose transporters facilitating increased glucose consumption, which may be utilized for targeted transport of therapeutics. In this paper we report the development and initial characterization of novel PAMAM–based nanocarrier for anticancer drugs, designed to specifically enter tumor cell subpopulations due to the enhanced glucose uptake. We were able to prove that the cytotoxic effect of PAMAM-dox-glc depends on the expression of functional GLUT1 surface protein, suggesting specific, transporter-dependent internalization as a main route of cellular uptake of glucose-conjugated PAMAM dendrimers. This indicates a great potential of GLUT-mediated transport of therapeutics into cancer cells and opens new roads for targeted delivery of anticancer drugs, which will be further assessed in our laboratory. Such an approach may contribute to the elaboration of efficient therapy utilizing specific, deregulated metabolism of cancer cells, at the same time decreasing the toxic side effects towards healthy tissues.

## Electronic supplementary material

ESM 1 (DOC 721 kb)

## References

[1] Forman D, Mathers C, Soerjomataram I, Bray F, Eser S, Rebelo M (2014). Cancer incidence and mortality worldwide: sources, methods and major patterns in GLOBOCAN 2012. Int J Cancer.

[2] Engstrøm M. J., Opdahl S., Hagen A. I., Romundstad P. R., Akslen L. A., Haugen O. A., Vatten L. J., Bofin A. M. (2013). Molecular subtypes, histopathological grade and survival in a historic cohort of breast cancer patients. Breast Cancer Research and Treatment.

[3] Oh TG, Wang SCM, Muscat GEO (2017). Therapeutic implications of epigenetic signaling in breast cancer. Endocrinology.

[4] Leclerc A-FF, Jerusalem G, Devos M, Crielaard J-MM, Maquet D (2016). Multidisciplinary management of breast cancer. Arch Public Heal.

[5] Gorzkiewicz M, Klajnert-Maculewicz B. Dendrimers as Nanocarriers for Anticancer Drugs. In: Sharma AK, Keservani RK, editors. Dendrimers for Drug Delivery. Apple Academic Press. 2018:327–74.

[6] De Jong WH, Borm PJA (2008). Drug delivery and nanoparticles:applications and hazards. Int J Nanomedicine.

[7] Ochubiojo M, Chinwude I, Ibanga E, Ifianyi S. Nanotechnology in drug delivery. Recent Adv Nov Drug Carr Syst. 2012:69–106.

[8] Singh R, LJ W, Lillard JW (2009). Nanoparticle-based targeted drug delivery. Exp Mol Pathol.

[9] Kakde D, Jain D, Shrivastava V, Kakde R, Patil AT (2011). Cancer therapeutics-opportunities, challenges and advances in drug delivery. JAPS..

[10] Klajnert B, Bryszewska M (2001). Dendrimers: properties and applications. Acta Biochim Pol.

[11] Gorzkiewicz M, Klajnert-Maculewicz B (2017). Dendrimers as nanocarriers for nucleoside analogues. Eur J Pharm Biopharm.

[12] Ekladious I, Colson YL, Grinstaff MW (2019). Polymer–drug conjugate therapeutics: advances, insights and prospects. Nat Rev Drug Discov.

[13] Kesharwani P, Jain K, Jain NK (2014). Dendrimer as nanocarrier for drug delivery. Prog Polym Sci.

[14] Fadaka A, Ajiboye B, Ojo O, Adewale O, Olayide I, Emuowhochere R (2017). Biology of glucose metabolization in cancer cells. J Oncol Sci.

[15] Liang Y, Feng Y, Liu W, Han J, Mai H, Kang L (2017). miR-30a-5p suppresses breast tumor growth and metastasis through inhibition of LDHA-mediated Warburg effect. Cancer Lett.

[16] Calvaresi EC, Hergenrother PJ (2013). Glucose conjugation for the specific targeting and treatment of cancer. Chem Sci.

[17] Marcinkowska M, Sobierajska E, Stanczyk M, Janaszewska A, Chworos A, Klajnert-Maculewicz B (2018). Conjugate of PAMAM dendrimer, doxorubicin and monoclonal antibody-trastuzumab: The new approach of a well-known strategy. Polymers (Basel).

[18] Hellemans J, Vandesompele J (2014). Selection of reliable reference genes for RT-qPCR analysis. Methods Mol Biol.

[19] Yabbarov NG, Posypanova GA, Vorontsov EA, Obydenny SI, Severin ES (2013). A new system for targeted delivery of doxorubicin into tumor cells. J Control Release.

[20] Honary S, Zahir F (2013). Effect of zeta potential on the properties of nano-drug delivery systems - a review (part 1 and 2). Trop J Pharm Res.

[21] Davis BG, Robinson MA (2002). Drug delivery systems based on sugar-macromolecule conjugates. Curr Opin Drug Discov Devel.

[22] Hartinger CG, Nazarov AA, Ashraf SM, Dyson PJ, Keppler K (2008). Carbohydrate-metal complexes and their potential as anticancer agents. Curr Med Chem.

[23] Klip A, Tsakiridis T, Marette A, Ortiz P A (1994). Regulation of expression of glucose transporters by glucose: a review of studies in vivo and in cell cultures. The FASEB Journal.

[24] Macheda ML, Rogers S, Best JD (2005). Molecular and cellular regulation of glucose transporter (GLUT) proteins in cancer. J Cell Physiol.

[25] Thorens B (2017). Glucose transporters in the regulation of intestinal, renal, and liver glucose fluxes. Am J Phys.

[26] Burant CF, Bell GI (1992). Mammalian facilitative glucose transporters: evidence for similar substrate recognition sites in functionally monomeric proteins. Biochemistry.

[27] Seino S, Bell GI, Fukumoto H, Seino Y, Imura H, Ojo O (2013). Characterization and expression of human HepG2/erythrocyte glucose-transporter gene. Diabetes.

[28] Augustin R (2010). The protein family of glucose transport facilitators: It’s not only about glucose after all. IUBMB Life.

[29] Douard V, Ferraris RP (2008). Regulation of the fructose transporter GLUT5 in health and disease. AJP Endocrinol Metab.

[30] Barron C, Tsiani E, Tsakiridis T (2012). Expression of the glucose transporters GLUT1, GLUT3, GLUT4 and GLUT12 in human cancer cells. BMC Proc.

[31] Li J, Ma F, Dang Q (2014). Glucose-conjugated chitosan nanoparticles for targeted drug delivery and their specific interaction with tumor cells. Front Mater Sci.

[32] Barbaro D, Di Bari L, Gandin V, Evangelisti C, Vitulli G, Schiavi E (2015). Glucose-coated superparamagnetic iron oxide nanoparticles prepared by metal vapour synthesis are electively internalized in a pancreatic adenocarcinoma cell line expressing GLUT1 transporter. PLoS One.

[33] Jiang X, Zhu L, Xin H, Sha X, Ren Q, Xie Y (2014). Nanoparticles of 2-deoxy-d-glucose functionalized poly(ethylene glycol)-co-poly(trimethylene carbonate) for dual-targeted drug delivery in glioma treatment. Biomaterials.

[34] Jiang Xinyi, Xin Hongliang, Gu Jijin, Du Fengyi, Feng Chunlai, Xie Yike, Fang Xiaoling (2014). Enhanced Antitumor Efficacy by d-Glucosamine-Functionalized and Paclitaxel-Loaded Poly(Ethylene Glycol)-Co-Poly(Trimethylene Carbonate) Polymer Nanoparticles. Journal of Pharmaceutical Sciences.

[35] Grover-McKay M, Walsh SA, Seftor EA, Thomas PA, Hendrix MJJC (1998). Role for glucose transporter 1 protein in human breast cancer. Pathol Oncol Res.

[36] Hu H, Geng XD, Gu N, Xiong F, Wang YF, Lin J (2011). Targeting Glut1-overexpressing MDA-MB-231 cells with 2-deoxy-d-g1ucose modified SPIOs. Eur J Radiol.

[37] Zhao J, Babiuch K, Lu H, Dag A, Gottschaldt M, Stenzel MH (2014). Fructose-coated nanoparticles: a promising drug nanocarrier for triple-negative breast cancer therapy. Chem Commun.

[38] Venturelli L, Nappini S, Bulfoni M, Gianfranceschi G, Dal Zilio S, Coceano G, et al. Glucose is a key driver for GLUT1-mediated nanoparticles internalization in breast cancer cells. Sci Rep. 2016;6:21629. 10.1038/srep21629 PMC4761954 26899926

[39] Ma Pengkai, Chen Jianhua, Bi Xinning, Li Zhihui, Gao Xing, Li Hongpin, Zhu Hongyu, Huang Yunfang, Qi Jing, Zhang Yujie (2018). Overcoming Multidrug Resistance through the GLUT1-Mediated and Enzyme-Triggered Mitochondrial Targeting Conjugate with Redox-Sensitive Paclitaxel Release. ACS Applied Materials & Interfaces.

[40] Martin H-JJ, Kornmann F, Fuhrmann GF (2003). The inhibitory effects of flavonoids and antiestrogens on the Glut1 glucose transporter in human erythrocytes. Chem Biol Interact.

[41] Araújo JR, Azevedo C, Correia-Branco A, Martel F, Guimarães JT, Keating E (2015). The chemopreventive effect of the dietary compound kaempferol on the MCF-7 human breast cancer cell line is dependent on inhibition of glucose cellular uptake. Nutr Cancer.

[42] George Thompson AM, Iancu CV, Nguyen TTH, Kim D, Choe JY (2015). Inhibition of human GLUT1 and GLUT5 by plant carbohydrate products; insights into transport specificity. Sci Rep.

[43] Krzeslak Anna, Wojcik-Krowiranda Katarzyna, Forma Ewa, Jozwiak Paweł, Romanowicz Hanna, Bienkiewicz Andrzej, Brys Magdalena (2012). Expression of GLUT1 and GLUT3 Glucose Transporters in Endometrial and Breast Cancers. Pathology & Oncology Research.

[44] Cantuaria Guilherme, Fagotti Anna, Ferrandina Gabriella, Magalhaes Albino, Nadji Merhad, Angioli Roberto, Penalver Manuel, Mancuso Salvatore, Scambia Giovanni (2001). GLUT-1 expression in ovarian carcinoma. Cancer.

