# Supplementary material for: Sugar Modification Enhances Cytotoxic Activity of PAMAM-Doxorubicin Conjugate in Glucose-Deprived MCF-7 Cells – Possible Role of GLUT1 Transporter
Source: Pharm Res. 2019 Jul 31;36(10):140. doi: 10.1007/s11095-019-2673-9 (PMC6669199; doi:10.1007/s11095-019-2673-9)
Supplement: Supplementary file 1 — (DOC 721 kb) [file 11095_2019_2673_MOESM1_ESM.doc]

**Supplementary Materials**

**Sugar modification enhances cytotoxic activity of PAMAM-doxorubicin conjugate in glucose-deprived MCF-7 cells – possible role of GLUT1 transporter**

Krzysztof Sztandera^1a^, Paula Działak^1a^, Monika Marcinkowska^1^, Maciej Stańczyk^2^, Michał Gorzkiewicz^1*^, Anna Janaszewska^1^, Barbara Klajnert-Maculewicz^1,3^

^1^ Department of General Biophysics, Faculty of Biology and Environmental Protection, University of Lodz, 141/143 Pomorska St, 90–236 Lodz, Poland

^2^ Department of Surgical Oncology, Cancer Center, Copernicus Memorial Hospital, 62 Pabianicka St, 93–513 Lodz, Poland

^3^ Leibniz–Institut für Polymerforschung Dresden e.V., 6 Hohe St, 01069 Dresden, Germany

***^1^H NMR analysis of dox-CAA (doxorubicin-*cis*-aconitic anhydride), glc-SA (glucose- succinic anhydride), PAMAM-dox conjugate and PAMAM-dox-glc conjugate***

1. ***^1^H NMR results for dox-CAA***

In the first step we prepared ^1^H NMR spectra (DMSO-d6, 300 MHz, ppm) for dox-CAA (Figure S1). We observed signals for aromatic (7.91 ppm belonging to the anthracene) and aliphatic (3.92 ppm for O–C**H_3_**, 4.86 ppm for C**H**–O, 4.53 ppm for C**H_2_**–OH, 5.18 ppm for C**H**–O from amine sugar ring) protons of dox. Upon cis-aconitic anhydride modification, additional signals at 6.58 and 6.18 ppm appeared, attributed to the protons (C**H**–COO–) of the cis-aconitic anhydride and the trans-aconitic anhydride linkages, respectively. The peak at 4.17 ppm belongs to the protons of –C**H_2_**COOH. The ratio of *cis*:*trans* forms equalled 1:2.


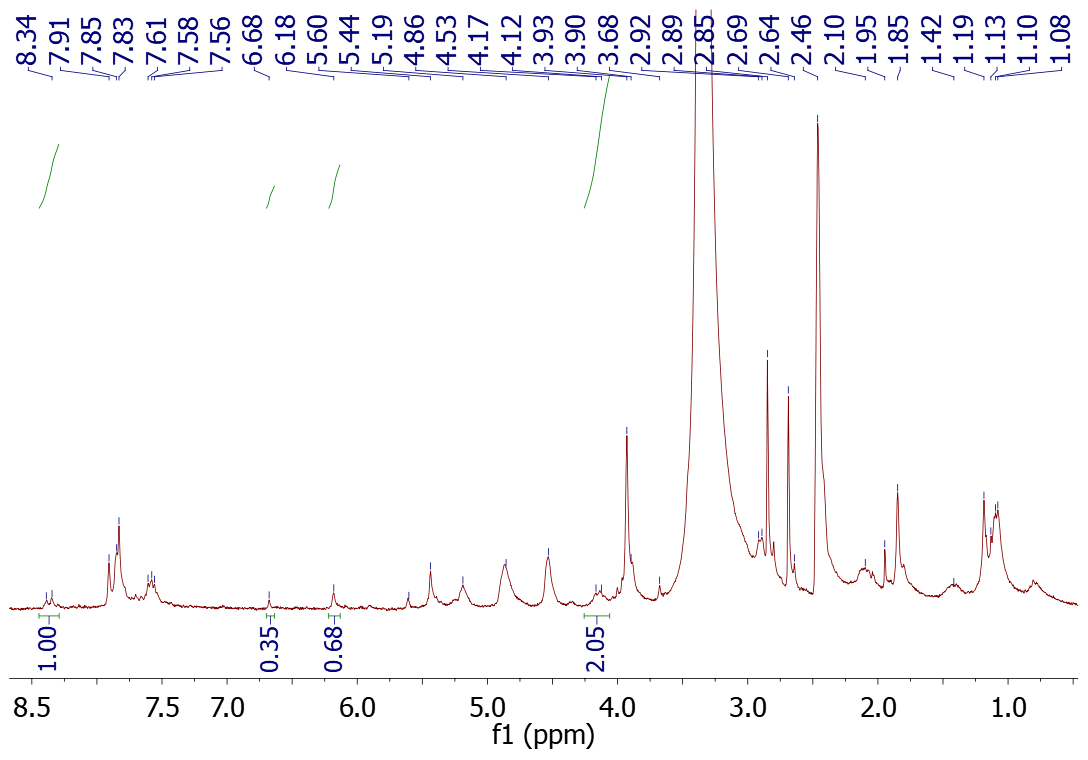


# *Figure S1. The ^1^H NMR spectrum of dox-CAA.*

# *^1^H NMR results for glc-SA*

In the second step we prepared ^1^H NMR spectra (DMSO-d6, 300 MHz, ppm) for glucose and glucose-SA (Figure S2 and S3). Analysing the spectra, it is very difficult to state clearly with which OH proton the reaction occurred. However, we observed the shift for C**H_2_** belonging to the succinic acid linked with glc at 2.37-2.35 ppm, indicating the bonding between C_6_-O**H** of glucose and C**H_2_** of succinic moiety.


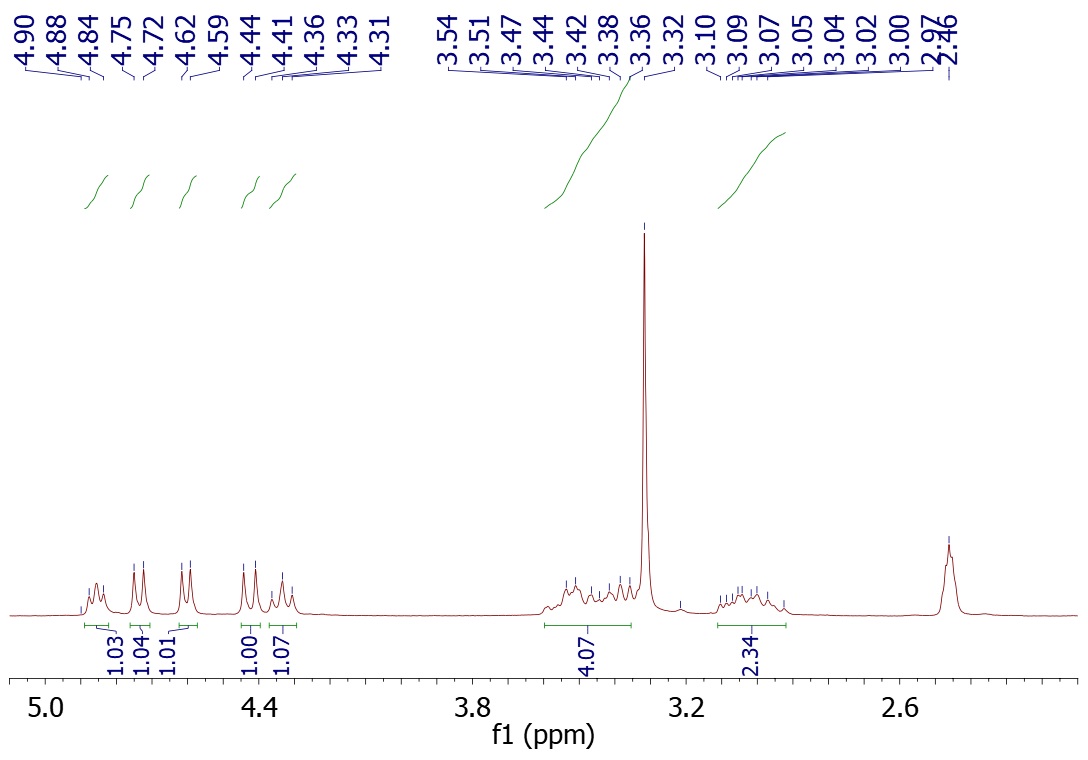


# *Figure S2. The ^1^H NMR spectrum of glucose*


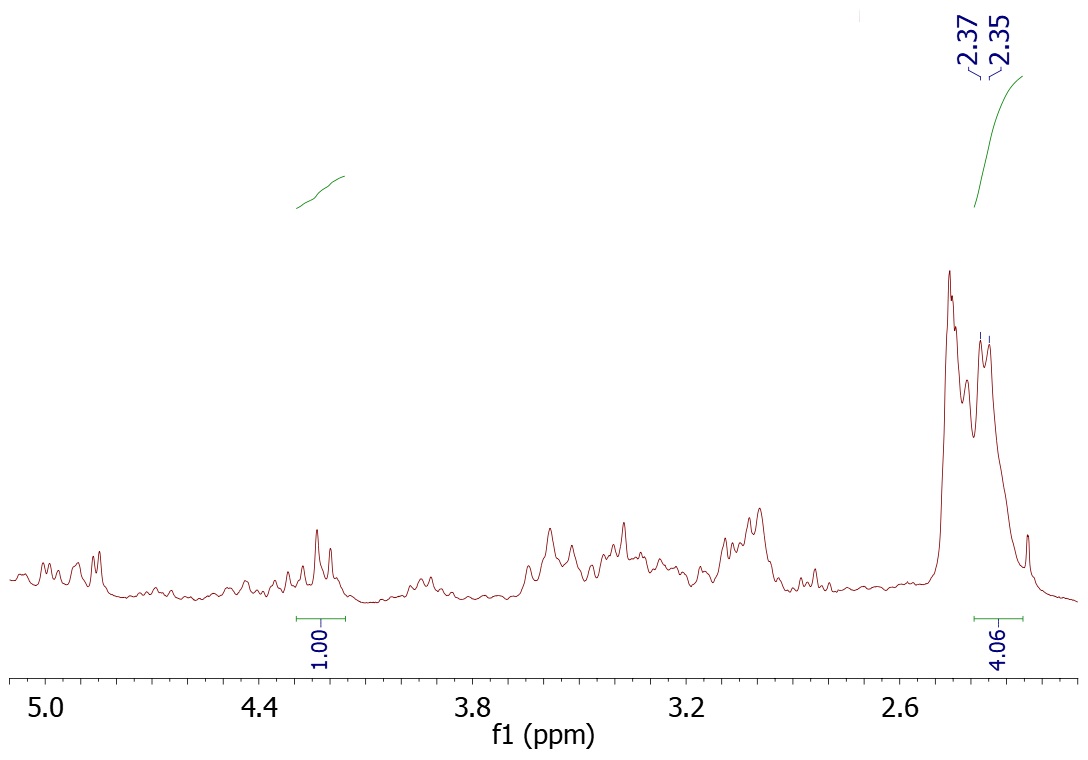


# *Figure S3. The ^1^H NMR spectrum of glc-SA.*

# *^1^H NMR results for PAMAM, PAMAM-dox and PAMAM-dox-glc*

In the Figure S4 we present ^1^H NMR spectra of the PAMAM G4-NH_2_ dendrimer (DMSO-d6, 300 MHz, ppm). The signals were as follows: 2.15 ppm (–C**H_2_**–C(O)–NH), 2.38 ppm (–C**H_2_**–N–), 2.51-2-59 ppm (–N–C**H_2_**–), 2.94–3.05 ppm (–C**H_2_**–NH_2_), 3.27–3.34 ppm (–C(O)NH–C**H_2_**), 7.96 ppm (–CON**H**–).


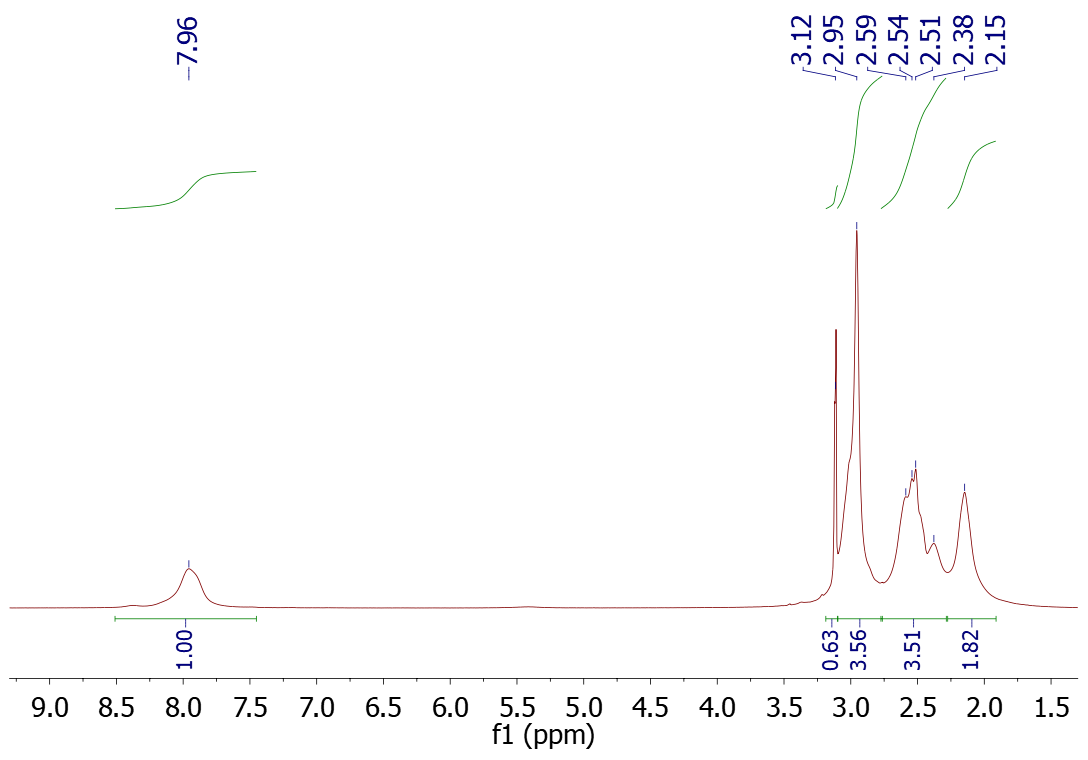


# *Figure S4. The ^1^H NMR spectrum of PAMAM dendrimer.*

^1^H NMR signals of the PAMAM-dox conjugate (DMSO-d6, 300 MHz, ppm; Figure S5) were as follows: 2.35–2.46 ppm (–C–C**H_2_**–C(O)–NH), 2.66 ppm (–N–C**H_2_**–CH_2_–NH), 2.85 ppm (–N–CH_2_–C**H_2_**–C(O)–), 2.94–3.05 ppm (–C**H_2_**–NH_2_), 3.27–3.34 ppm (–C(O)NH–C**H_2_**), 8.21 and 8.42 ppm (–C(O)–N**H**–). We observed the shifts of signals attributed to the protons of *cis*-aconitic anhydride linkages (6.17 and 6.05 ppm) and new signal at 3.94 ppm belonging to the protons of –C**H_2_**–C(O)–NH–. The PAMAM:dox (N**H**:C**H**) bonding indicated that the reaction has occurred in a 1:1 ratio.


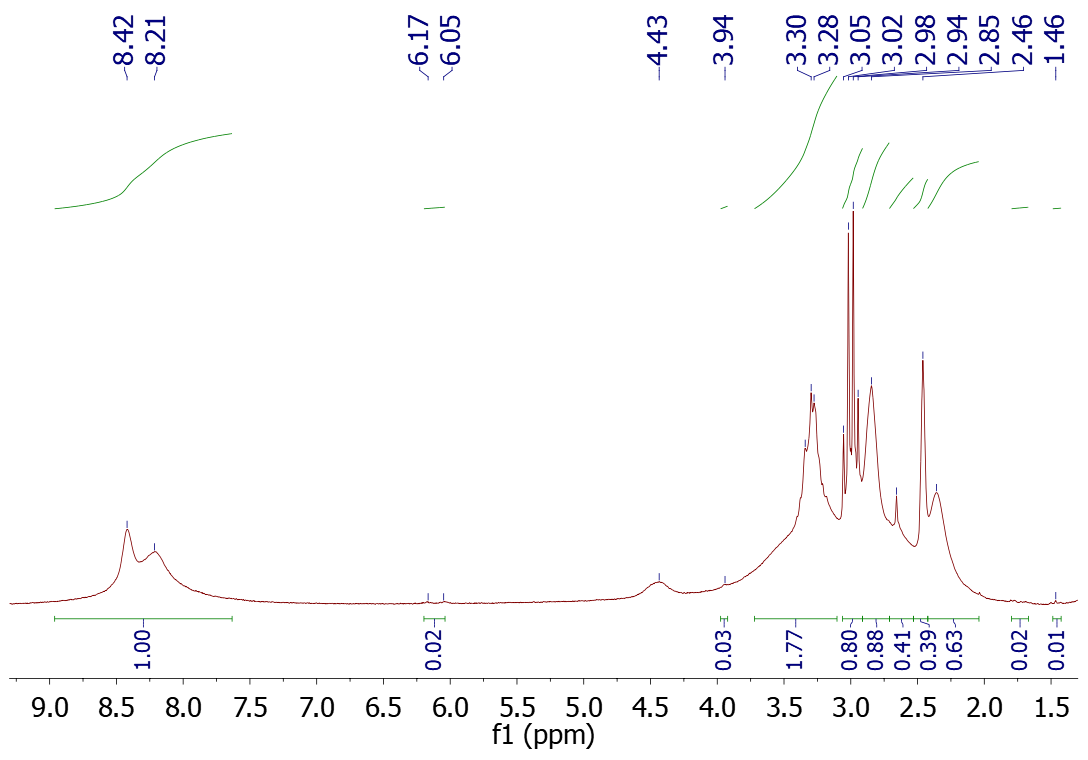


# *Figure S5. The ^1^H NMR spectrum of PAMAM-dox conjugate.*

^1^H NMR signals of the PAMAM-dox-glc conjugate (DMSO-d6, 300 MHz, ppm; Figure S6) were as follows: 2.35–2.46 ppm (–C–C**H_2_**–C(O)–NH), 2.66 ppm (–N–C**H_2_**–CH_2_–NH), 2.85 ppm (–N–CH_2_–C**H_2_**–C(O)–), 2.94–3.05 ppm (–C**H_2_**–NH_2_), 3.27–3.34 ppm (–C(O)–NH–C**H_2_**), 8.18 and 8.40 ppm (–CO–N**H**–). We observed the signals attributed to the protons of cis-aconitic anhydride linkages (6.17 and 6.05 ppm) and new signals at 4.20–4.23 and 4.86–4.90 ppm belonging to the protons of –C–O**H** group of glucose. The PAMAM:glc (N**H**:C_6_-O**H**) bonding indicated that the reaction has occurred in a 1:1 ratio.


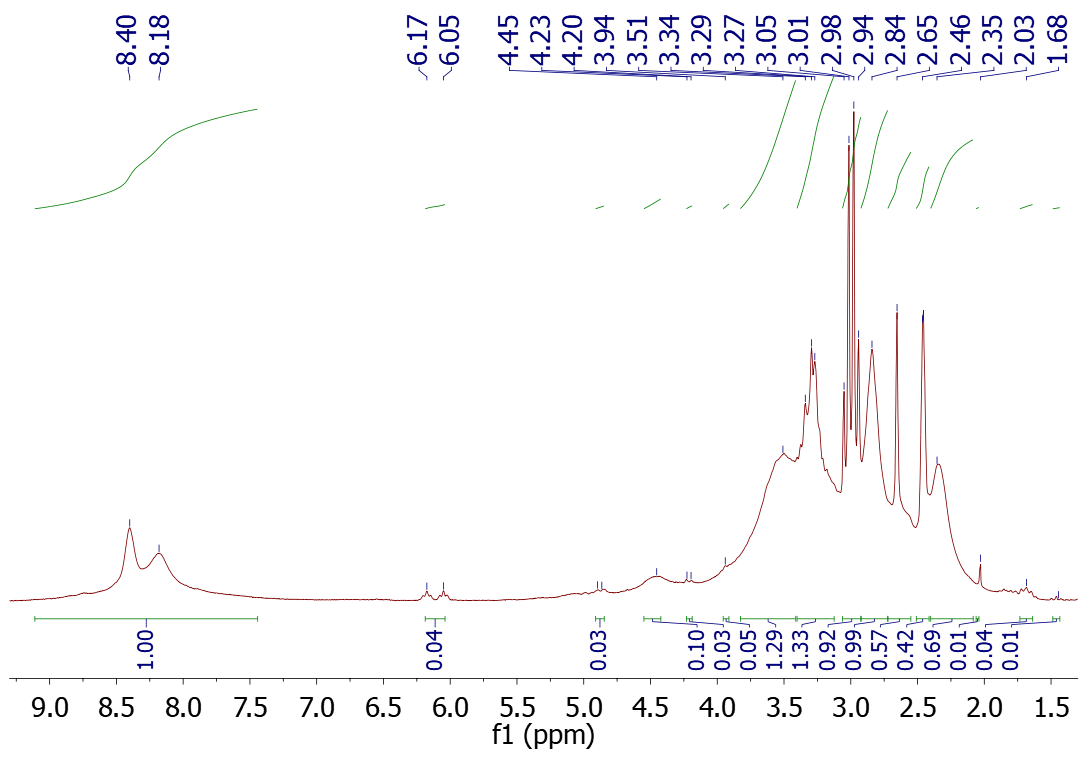


# *Figure S6. The ^1^H NMR spectrum of PAMAM-dox-glc conjugate.*

***FT-IR spectra of the products containing dox***

FT-IR spectra were used to confirm the structure of obtained compounds. The signals that appeared at approximately 1548 and 1558 cm^−1^ in dox-CAA were assigned to the characteristic signals of the amide bond. In the second and the third spectrum, the signal of –CONH­– stretch was observed at 1668 and 1566 cm^−1^, indicating the grafting of carboxyl group on the PAMAM dendrimer surface. FT-IR spectra of dox-CAA, PAMAM-dox and PAMAM-dox-glc are given in Figure 7.


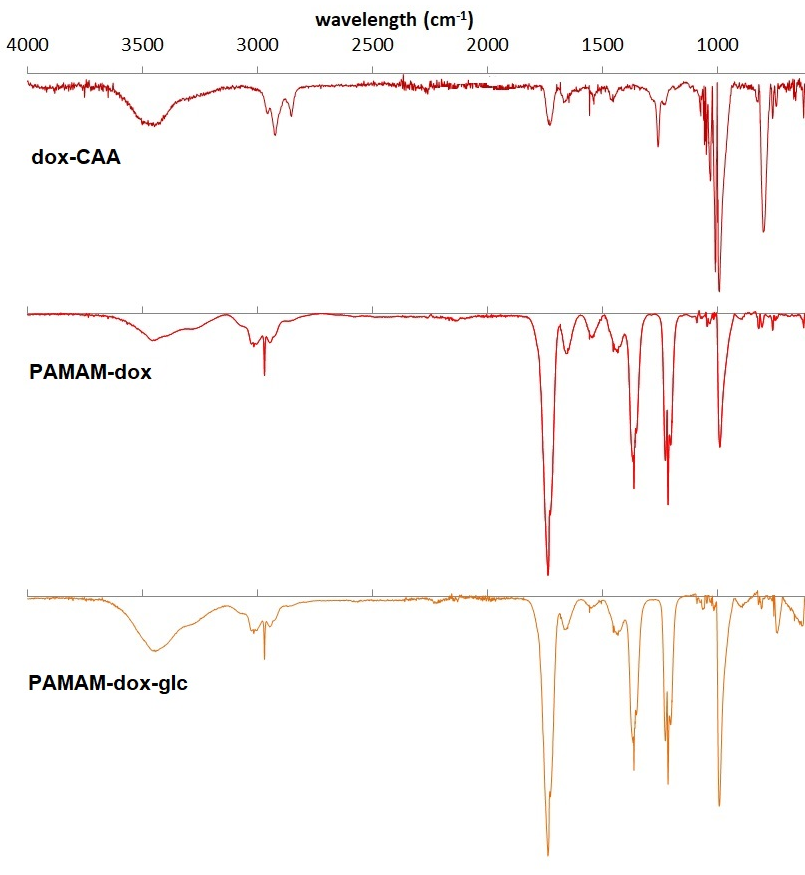


# *Figure S7. The FT-IR spectra of dox-CAA, PAMAM-dox and PAMAM-dox-glc conjugates.*
